# A Rare Variation in the Origin and Course of the Artery of Penis

**DOI:** 10.1155/2014/193194

**Published:** 2014-02-12

**Authors:** Satheesha B. Nayak, Naveen Kumar, Jyothsna Patil, Surekha D. Shetty, Srinivasa Rao Sirasanagandla, Swamy Ravindra

**Affiliations:** Department of Anatomy, Melaka Manipal Medical College (Manipal Campus), International Centre for Health Sciences, Manipal University, Udupi District, Madhavagar, Manipal , Karnataka 576 104, India

## Abstract

Vascular variations of the penis are very rare. Awareness of its variations is of utmost importance to the urologists and radiologist dealing with the reconstruction or transplants of penis, erectile dysfunctions, and priapism. We report an extremely rare variation of the artery of the penis and discuss its clinical importance. The artery of the penis arose from a common arterial trunk from the left internal iliac artery. The common trunk also gave origin to the obturator and inferior vesical arteries. The artery of the penis coursed forward in the pelvis above the pelvic diaphragm and divided into deep and dorsal arteries of the penis just below the pubic symphysis. The internal pudendal artery was small and supplied the anal canal and musculature of the perineum. It also gave an artery to the bulb of the penis.

## 1. Introduction

The artery of the penis is the distal continuation of the internal pudendal artery after the origin of its perineal branch. It runs anteriorly below or above the inferior fascia of urogenital diaphragm to reach the area just below the inferior pubic ligament, where it terminates by dividing into deep and dorsal arteries of the penis [1]. Artery of the penis may rarely arise directly from the internal iliac artery and continue as the deep artery of the penis when the dorsal artery of the penis is a branch of internal iliac artery or the inferior epigastric artery [2]. Knowledge of variations in the origin and course of the artery of the penis is important to radiologists, surgeons, and urologists. We report an extremely rare variation of the artery of the penis and to the best of our knowledge this is the first report on such a variation. The clinical and surgical implication of the variation is discussed.

## 2. Case Report

**Figure 1:**
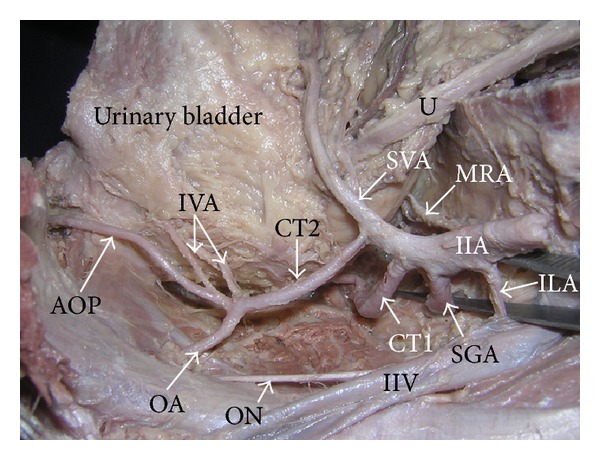
Superior view of the left hemipelvis. (AOP: artery of penis, CT1: common trunk 1, CT2: common trunk 2, IIA: internal iliac artery, IIV: internal iliac vein, ILA: iliolumbar artery, IVA: inferior vesical arteries, MRA: middle rectal artery, OA: obturator artery, ON: obturator nerve, SGA: superior gluteal artery, SVA: superior vesical artery, and U: ureter.)

**Figure 2:**
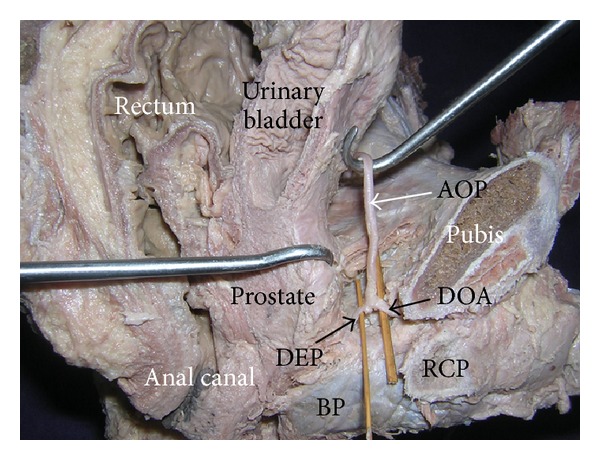
Sagittal section of the pelvis showing the sectional views of rectum, canal, urinary bladder, prostate, and the pubis. (AOP: artery of penis, BP: bulb of penis, DEP: deep artery of the penis, DOA: dorsal artery of the penis, and RCP: right crus of the penis.)

During dissection classes for undergraduate medical students, a rare variation in the origin and course of the artery of the penis was noted. The variation was found in an adult male cadaver aged approximately 70 years. The left internal iliac artery did not divide into anterior and posterior divisions. The main trunk of the internal iliac artery gave iliolumbar, lateral sacral, superior gluteal, middle rectal, and superior vesical arteries. In addition to these arteries two common trunks arose from it. The first common trunk bifurcated into inferior gluteal and internal pudendal arteries, whereas the second common trunk gave two inferior vesical arteries, obturator artery, and the artery of penis (Figure 1). The artery of the penis coursed forwards between the bladder and lateral pelvic wall until the pubis. Thereafter it passed below the puboprostatic ligaments and divided into deep and dorsal arteries of the penis just behind the lower border of the pubic symphysis (Figure 2). The dorsal artery of penis passed between the lower border of the pubic symphysis and transverse perineal ligament to enter the dorsum of the penis. The deep artery pierced the perineal membrane and entered the anterior part of the left crus of the penis. The left internal pudendal artery was reduced in size and it supplied the musculature of the perineum and the anal canal. It also supplied the bulb of the penis.

## 3. Discussion

A detailed knowledge of origin, course, and distribution of the vessels of the penis is essential during the planning, management, or surgical treatment of erectile dysfunctions and trauma of the penis. Internal iliac artery embolization is standard selective technique in arresting the bleeding from the penis and perineal region. Instead of this procedure, a superselective embolization of the internal pudendal artery with a stainless steel mini coil can be done to preserve the blood supply from the uninjured branches of the internal iliac artery [3]. However, when the artery of the penis has a variant origin as in the current case, the superselective embolization of internal pudendal artery might not give the desired outcome. Transplantation of the penis from a donor includes anastomosis of urethra, corpus spongiosum, and corpus cavernosum. It also involves suturing of deep dorsal vein, dorsal artery, dorsal nerve, and superficial dorsal vein [4]. Knowledge of possibility of a variation as reported in this case might significantly add to the success of the technique. Erectile dysfunction is one of the conditions that affect the social and psychological status of a male. 10% of the men between 40 and 60 years can expect to suffer from erectile dysfunction [5]. Arterial malformation alone or in combination with other causes has been observed in over 60% of patients with primary erectile dysfunction [6]. The arteriographic assessment of morphological changes in internal pudendal and penile arteries is the most crucial step in the diagnosis of vascular erectile dysfunction, especially before undertaking invasive therapeutic procedures. Knowledge of current variation might be handy in performing radiological diagnostic and invasive therapeutic procedures.

Traumatic laceration of penile arteries may result in high flow priapism caused by a pathologically increased arterial flow to the cavernous bodies. Clinically, it is identified as a persistent painless erection with a flaccid glans that results within hours or days after blunt perineal trauma [7–9]. Patient's physical examination followed by corporeal blood gas analysis and color Doppler ultrasound or arteriogram is very useful to make a diagnosis in such a case [9]. The condition can be treated in various ways such as transcatheter embolization with autologous clots, microcoils or gelatin sponges, shunt operations, and open surgery [8]. All these techniques might face difficulties when the artery of the penis has variant origin and course as the one being reported here. Other variations in the branching pattern of the internal iliac artery mentioned in this report are quite common and have been reported earlier [10, 11].

As per our knowledge, this is the first report on combined origin of artery of the penis, obturator artery, and inferior vesical arteries from a common trunk above the pelvic diaphragm. What makes the case unique is the forward course of artery of the penis in relation to the pelvic surface of the levator ani muscle, prostate, and puboprostatic ligaments. Its iatrogenic injuries are possible in any procedure involving prostate and bladder. The artery might get injured in pelvic fractures as well. Hence, this report might be useful for the radiologists, orthopaedic surgeons, and urologists working in and around the pelvic cavity.
